# Comprehensive Analysis of Colorectal Cancer Immunity and Identification of Immune-Related Prognostic Targets

**DOI:** 10.1155/2022/7932655

**Published:** 2022-03-30

**Authors:** Huijuan Wen, Fazhan Li, Ihtisham Bukhari, Yang Mi, Chenxu Guo, Bin Liu, Pengyuan Zheng, Simeng Liu

**Affiliations:** ^1^Henan Key Laboratory of Helicobacter Pylori & Microbiota and Gastrointestinal Cancer, Marshall Medical Research Center, The Fifth Affiliated Hospital of Zhengzhou University, Zhengzhou 450002, China; ^2^Academy of Medical Science, Zhengzhou University, Zhengzhou, China; ^3^Department of Gastroenterology, The Fifth Affiliated Hospital of Zhengzhou University, Zhengzhou, 450052 Henan, China

## Abstract

Colorectal cancer (COAD) is ranked as the third most common cancer and second in terms of cancer-related deaths worldwide. Due to its poor overall survival and prognosis, the incidents of COAD are significantly increasing. Although treatment methods have greatly been improved in the last decade, it is still not good enough to have satisfactory treatment outcomes. In recent years, immunotherapy has been successful to some extent in the treatment of many cancers but still, many patients do not respond to immunotherapy. Therefore, it is essential to have a deeper understanding of the immune characteristics of the tumor microenvironment and identify meaningful immune targets. In terms of immune targets, COAD has been poorly explored; thus, in the current study, based on the immune cell infiltration score and differentially expressed genes, COAD tumors were classified into hot and cold tumors. The Least Absolute Shrinkage and Selection Operator (LASSO) regression analysis was used to identify hub genes, construct a prognostic model, and screen potential immune targets. In total, 12 genes (*CLK3*, *CYSLTR2*, *GJA10*, *CYP4Z1*, *FAM185A*, *LINC00324*, *EEF1A1P34*, *EEF1B2P8*, *PTCSC3*, *MIR6780A*, *LINC01666*, and *RNU6.661P*) differentially expressed between hot and cold tumors were screened out. Among them, *CYSLTR2* was considered as a potential candidate gene, because it showed a significant positive correlation with immune cell infiltration and immune checkpoints (*PDCD1*, *CD274*, and *CTLA4*). Finally, we constructed and validated a new prognostic model for COAD showing 0.854 AUC for the ROC curve, and these results provide sufficient potential to choose *CYSLTR2* as an important immune target for the prognosis of COAD.

## 1. Introduction

Colorectal cancer (COAD) is ranked as the third most common malignancy, affecting approximately a million patients every year worldwide [1]. Although the overall survival of the patients diagnosed at early stages has increased due to surgical and therapeutic advancements [2, 3], the recurrence rate of patients with stages I-III and stage IV colon cancer is still 30% and 65%, respectively [4]. Presently, TNM staging is considered as a standard for the prognostic evaluation of colon cancer. However, the prognostic values always vary in different patients even if the TNM staging is performed by similar methods [5, 6], that is probably due to different immune targets. Therefore, it is highly needed to find new diagnostic and prognostic markers for COAD. Many recent studies have pointed out that the progression of colon cancer is affected by the immune microenvironment [7], which makes immunotherapy a potential treatment option for patients with COAD.

The tumor microenvironment contains not only tumor cells, but also many mesenchymal cells (tumor-associated fibroblasts), macrophages, and many remotely recruited tumor cells such as infiltrating immune cells and bone marrow-derived cells [8]. A large number of studies have shown that the microenvironment plays a crucial role in the tumor progression [9]. In the tumor microenvironment, tumor cells can directly invade the surrounding or other tissues through blood or lymphatic metastasis. In response to tumor cell invasion, the host tissues produce cytokines, cytokine receptors, or other factors to directly or indirectly regulate tumor cell proliferation [10]. Recently, according to the infiltration of immune cells in the tumor microenvironment, cancers can be classified into immunologically active “inflamed” (hot) tumors and inactive “noninflamed” tumors (cold) tumors [11]. The level of immune cell infiltration in cold tumors is relatively low, and generally speaking, due to this reason, satisfactory results cannot be achieved for chemotherapy and immunotherapy [12]. Dividing samples into cold and hot tumors based on different levels of immune cell infiltration for comparison is very important for identifying immune-related prognostic targets.

In the current study, based on the immune score, we classified COAD into hot and cold tumor groups. Then, differential analyses of hot and cold tumors were performed and further identified 12 hub genes via LASSO regression analysis. We constructed and validated a model for genes related to the prognosis of COAD. Then, the genes associated with the immune-related pathways were downloaded; we determined the importance of *CYSLTR2* in the prognosis of immunity and COAD. In general, our prognostic model can successfully predict the prognosis of COAD, and *CYSLTR2* could be a novel potential prognostic target for COAD immunotherapy.

## 2. Methods and Materials

### 2.1. Source of Data

**Figure 1:**
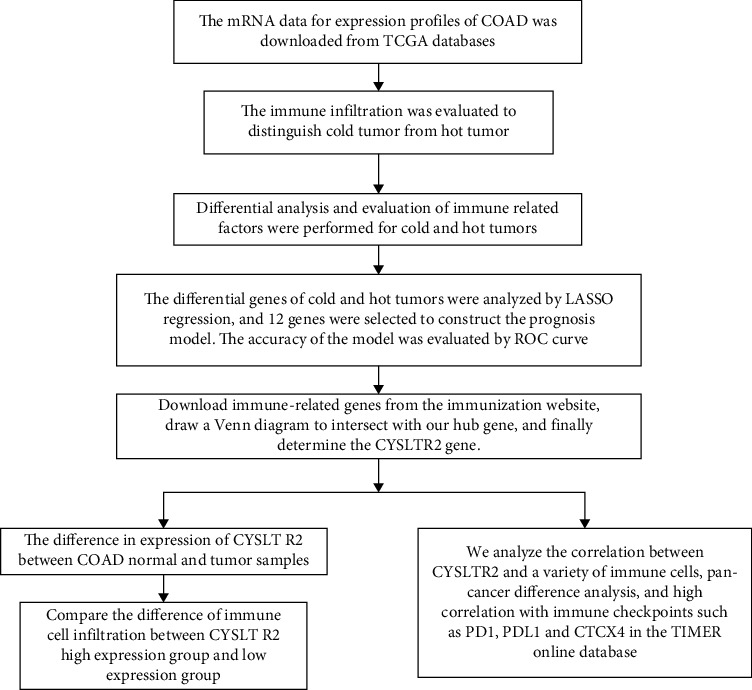
The general research design and flow of this study.

RNA profiles including HTseq-count and fragments per kilobase of exon per million read mapped (FPKM) of 459 primary COADs were downloaded from TCGA database (http://tcga.cancer.gov/dataportal) [13]. The complete clinical information and survival rate of the patients were also downloaded. The overall experimental design flow chart is shown in Figure 1.

### 2.2. Cell Consensus Clustering

Consensus clustering provides quantitative and visual estimates of unsupervised classes in a dataset [14]. We used the “Consensus Cluster Plus” package (http://www.bioconductor.org/) to perform the immune infiltration clustering based on single-sample gene set enrichment analysis (ssGSEA) in the R environment. Different clusters were displayed in the form of heat maps, and the tumors with a high degree of immune cell infiltration were defined as hot tumors while the tumor with low immune cell infiltration was defined as cold tumors.

### 2.3. Differently Expressed Genes between Cold and Hot Tumors

We analyzed the genetic differences between the two groups of hot and cold tumors and drew a heat map and volcano map to show the differentially expressed genes, which was based on ∣log2FC | >1, adjusted *P* value < 0.05. The tumor purity (TP), ESTIMATE score (ES), immune score (IS), and stromal score (SS) of each COAD cluster were calculated using the ESTIMATE algorithm in the package R software.

### 2.4. Expression of Immune-Related Factors in Different Clusters of Cold and Hot Tumors

We compared the expression levels of the immune-related factors (chemokines, antigen-presenting proteins, cytokines, and immune checkpoints) in cold and hot tumor samples by using the “ggplot2” package.

### 2.5. LASSO Regression Analysis

The univariate Cox regression analysis was performed to find the effect of each gene on the overall survival of the COAD patients. The relevant genes with *P* < 0.01 were considered for LASSO regression analysis. Then, we constructed a prognostic model for the hub gene. Kaplan-Meier curve analysis was performed to verify the relationship between risk score and survival rate (separated by median value). To further verify the accuracy of the prognostic model, the area under the curve (AUC) of the ROC curve was calculated.

### 2.6. Rescreening of Immune-Related Genes

Based on the level of immune cell infiltration, the tumors were divided into hot and cold tumors. To further screen out the hub gene and the immune-related genes, a total of 1811 immune-related genes were downloaded from the https://www.immport.org/. A Venn diagram was drawn for the identified hub genes, and the difference of *CYSLTR2* expression between tumor and normal samples was especially analyzed.

### 2.7. The Relationship between *CYSLTR2* and Immune Cells

The CIBERSORT package was used to identify the degree of infiltration of the 22 immune cells in different samples [15]. Furthermore, the degree of immune cell infiltration was compared between the groups having expression and low expression of *CYSLTR2*, and the difference of immune cell infiltration was presented as a violin chart. The “perm” was set at 100, and the samples with *P* < 0.05 in CIBERSORT were used in further analysis. Tumor Immune Estimation Resource (TIMER, http://cistrome.shinyapps.io/timer) [16] was used to compare the correlation between *CYSLTR2* and different immune checkpoints (*PDCD1*, *CD274*, and *CTLA4*), and then, the expression of *CYSLTR2* in pan-cancer including 32 cancers was also compared.

### 2.8. Statistical Analysis

All the analyses were performed using R version 3.6.3. Data were normalized using the “Sva” package [17]. The difference in the infiltration of immune cells between hot and cold tumors was analyzed using the Wilcoxon test. The survival analysis was analyzed by R package “survival,” while AUC was analyzed by R package “survivalROC.” The median value was set as the cut-off. The “glmnet” R package was used for LASSO analysis, and *P* value ≤ 0.05 was considered significant.

## 3. Results

### 3.1. Evaluation and Clustering of Immune Infiltration between Different Samples

**Figure 2:**
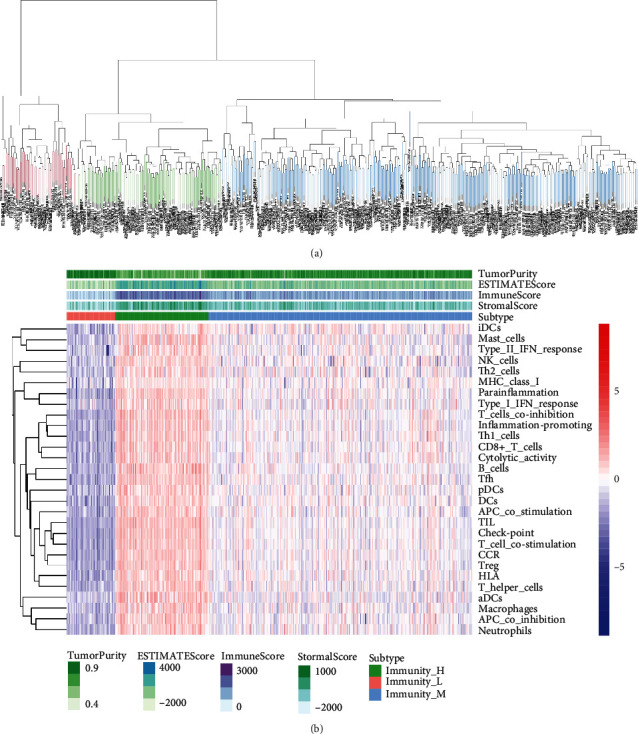
Clustering and immune assessment of different samples. (a) All tumor samples are clustered according to different immune cell infiltration. (b) The heat map shows the difference of immune cells in different samples, in which green is the high immune infiltration group, blue is the middle group, and red is the low group.

First, we assessed the pattern of immune cell infiltration and clustered the different COAD samples into three clusters based on ssGSEA (Figure 2(a)). Then, we drew a heat map to show the difference in the distribution of different immune cells, TP, ES, IS, and SS in different clusters. In general, we divided all of the COAD tumor samples into three groups: high, medium, and low immune cell infiltration. The corresponding heat map clearly shows the gradual decrease of immune cell infiltration in the three groups (Figure 2(b)).

### 3.2. Analysis of the Difference between Hot and Cold Tumors

**Figure 3:**
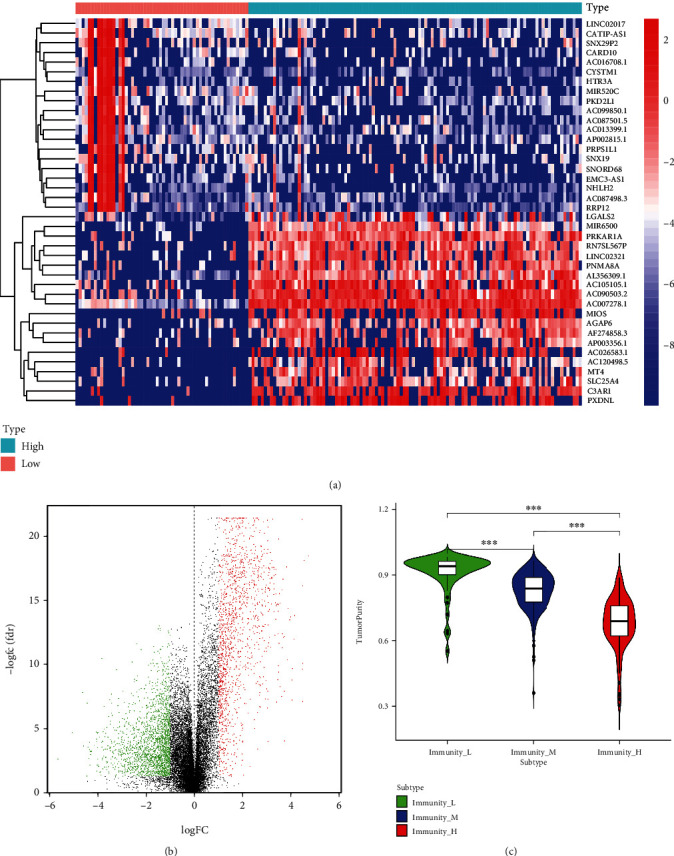
Analysis of the difference between hot and cold tumors. (a) A heat map and (b) volcano map for the DEGs between hot and cold tumors. (c) TP difference between cold tumors and hot tumors.

We clustered COAD tumor samples according to their difference in score of immune cell infiltration. Then further, the group with a high degree of immune infiltration was defined as the hot tumor, and the group with a low degree of immune cells was defined as the cold tumor. The volcano and heat maps show the difference in the gene expression between hot and cold tumors (Figures 3(a) and 3(b)). In total, 1443 upregulated and 2035 downregulated differentially expressed genes (DEGs) were screened out. Finally, we compared the TP between cold tumors and hot tumors (Figure 3(c)).

### 3.3. Differential Expression of Immune-Related Genes in Cold and Hot Tumors

**Figure 4:**
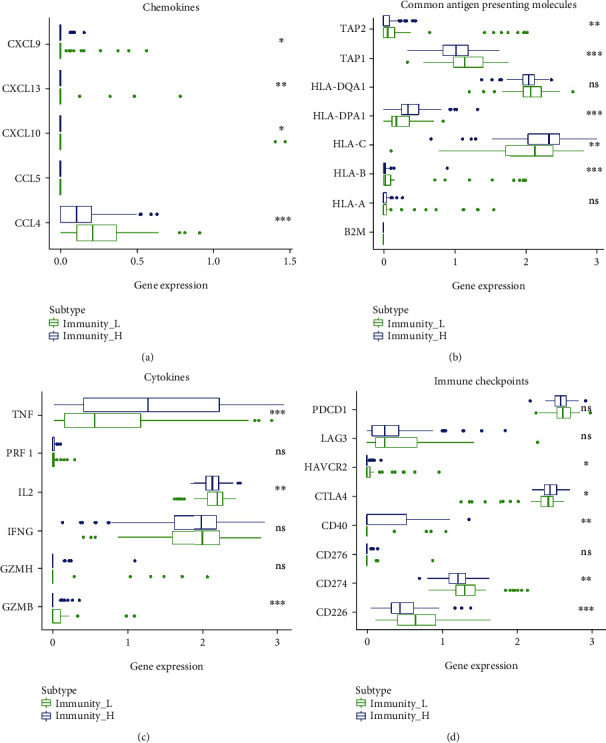
Box plot for the expression of immune-related genes between cold tumor and hot tumor. (a–d) The expression levels of multiple immune genes among the cold tumor and hot tumor. ^∗^*P* < 0.05, ^∗∗^*P* < 0.01, ^∗∗∗^*P* < 0.001, and ^∗∗∗∗^*P* < 0.0001.

The relationships between expression of immune checkpoints (*CD226*, *CD274*, *CD276*, *CD40*, *CTLA4*, *HAVCR2*, *LAG3*, and *PDCD1*), common antigen-presenting molecules (*B2M*, *HLA-A*, *HLA-B*, *HLA-C*, *HLA-DPA1*, *HLA-DQA1*, *TAP1*, and *TAP2*), cytokines (*GZMB*, *GZMH*, *IFNG*, *IL2*, *PRF1*, and *TNF*), and chemokines (*CCL4*, *CCL5*, *CXCL10*, *CXCL13*, and *CXCL9*) were analyzed. The results showed that the expression of some immune-related molecules in the hot tumor group was significantly higher than that in cold tumors, including common antigen-presenting molecules: *HLA-C* and *HLA-DPA1*, in immune checkpoints: *CTLA4* and *CD40*, and in cytokines: *TNF* (Figures 4(a)–4(d)).

### 3.4. Build a Prognostic Model Based on DEGs

**Figure 5:**
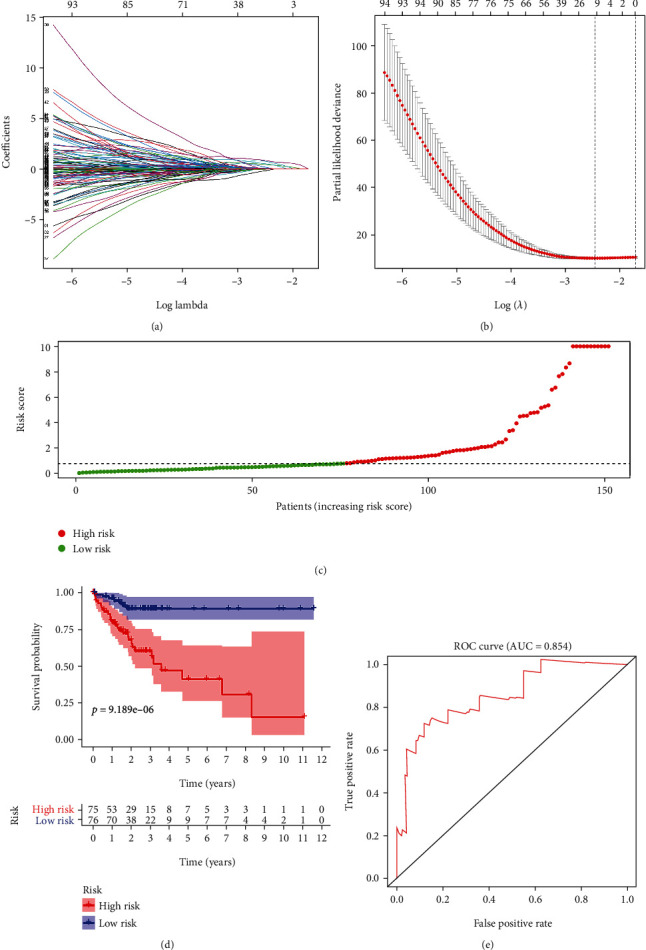
Constructing a prognostic model. (a–c) Determination of the number of factors by the LASSO analysis and the distribution of risk score. (d) Survival curves of the high-risk group and the low-risk group. (e) ROC curve to show the accuracy of the prediction model.

We incorporated the differential genes between cold and hot tumors into the LASSO analysis and identified 12 hub genes (*CLK3*, *CYSLTR2*, *GJA10*, *CYP4Z1*, *FAM185A*, *LINC00324*, *EEF1A1P34*, *EEF1B2P8*, *PTCSC3*, *MIR6780A*, *LINC01666*, and *RNU6.661P*), which were also included in the classifier (Figures 5(a)–5(c)). Kaplan-Meier analysis showed that the high-RS group showed a poor overall survival rate than the low-RS group (Figure 5(d)). The ROC curve was used to show the predictive power of the prognostic model (Figure 5(e)), and the AUC was detected as 0.854 in 5 years.

### 3.5. Screening for Immune-Related Genes

**Figure 6:**
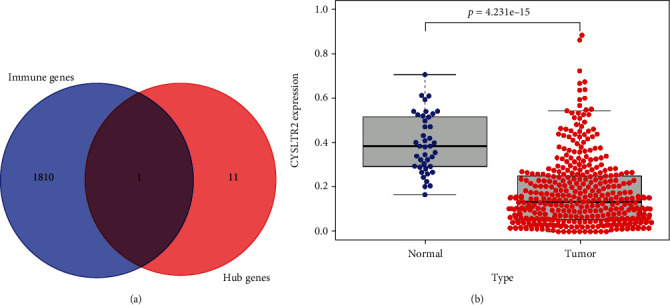
Screening of hub gene for immunity. (a) The Venn diagram shows that *CYSLTR2* is a possible immune-related gene. (b) The difference in expression of *CYSLTR2* gene in COAD normal and tumor tissues.

A total of 1811 immune-related genes were downloaded from an online database, and a total of 12 hub genes were intersected (Figure 6(a)). Then, in TCGA-COAD, the *CYSLTR2* showed low expression in colon tumors as compared to the corresponding normal tissues (Figure 6(b)), suggesting that it may act as a tumor suppressor.

### 3.6. The Relationship between *CYSLTR2* and Immune Cells

**Figure 7:**
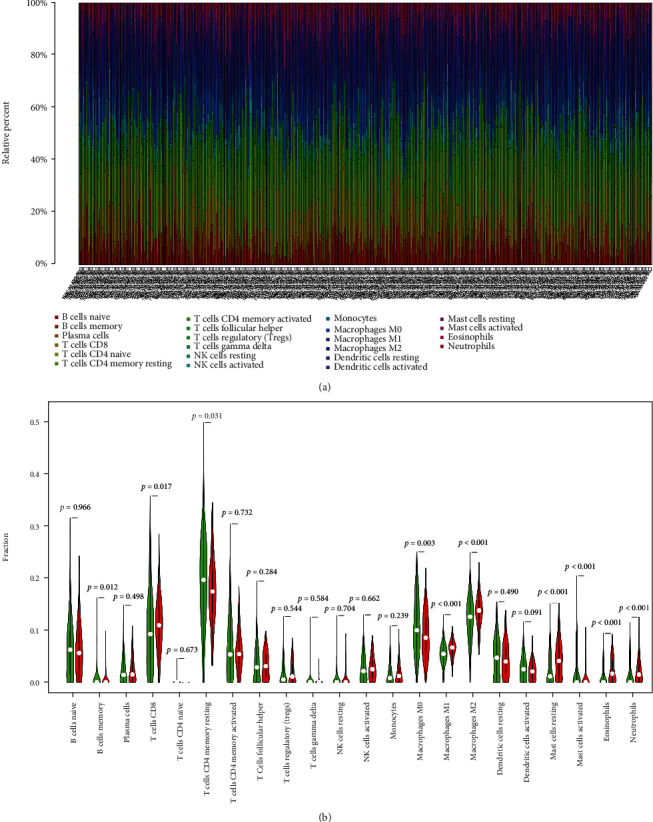
The relationship between *CYSLTR2* and immune cells. (a) Differences in the content of immune cells in different tumor samples. (b) Immune cell infiltration difference between the *CYSLTR2* high-expression group and the low-expression group. Red indicates the *CYSLTR2* high-expression group, and green indicates the *CYSLTR2* low-expression group.

**Figure 8:**
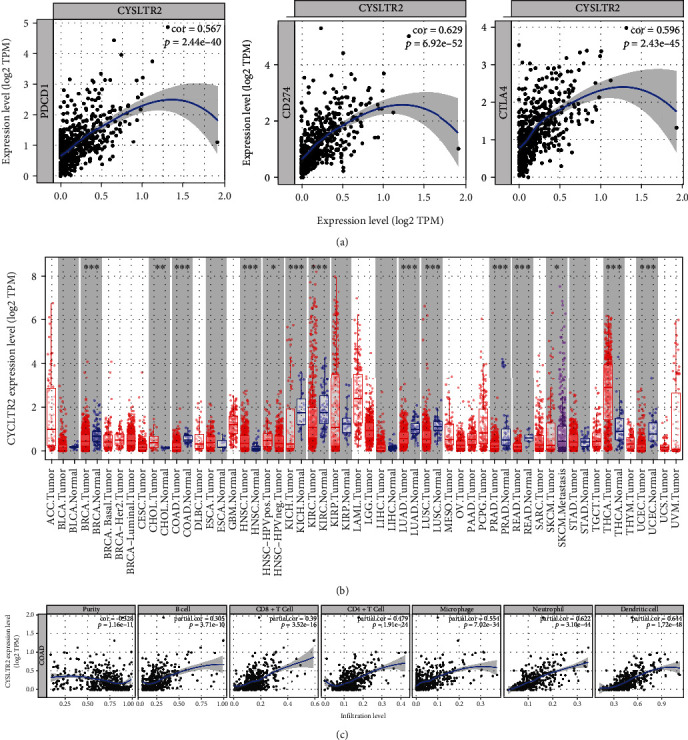
The relationship between *CYSLTR2* and different immune checkpoints and immune cells. (a) The relationship between *CYSLTR2* and different immune checkpoints (*PDCD1*, *CD274*, and *CTLA4*). (b) The difference in expression of *CYSLTR2* between cancerous tissues and adjacent normal tissues in a variety of cancers. (c) In COAD, *CYSLTR2* has a very significant correlation with TP, B cell, CD8+ T cell, CD4+ T cell, macrophage, neutrophil, and dendritic cell.

Based on the expression level of the *CYSLTR2*, tumor samples were divided into high-expression and low-expression groups. The proportion of 22 immune cells in different samples is shown in Figure 7(a). The difference in immune cell infiltration between the *CYSLTR2* high-expression and the low-expression groups is presented as violin chart (Figure 7(b)). We found that the high-expression group has significantly high levels of T cell CD8, macrophage M1, macrophage M2, mast cells, eosinophils, and neutrophils. To further evaluate the correlation between *CYSLTR2* and immune cells, we first analyzed the relationship between *CYSLTR2* and different immune checkpoints (*PDCD1*, *CD274*, and *CTLA4*) (Figure 8(a)) showing that *PDCD1*: cor = 0.567, *P* = 2.44∗10‐40; *CD274*: cor = 0.629, *P* = 6.92∗10‐52; and *CTLA4*: cor = 0.596, *P* = 2.43∗10‐45. Furthermore, we analyzed the expression of *CYSLTR2* in different tumors and adjacent tissues and found low expression of *CYSLTR2* in most of the solid tumors (Figure 8(b)). Lymphocytes' infiltration in the tumors is also an independent predictor of survival. Thus, exploring the correlation between genes and immune cells could help to screen suitable immune-related prognostic targets [18, 19]. Finally, we found the expression of *CYSLTR2* was *highly* correlated with the TP, B cell, CD8+ T cell, CD4+ T cell, macrophage, neutrophil, and dendritic cell in COAD (Figure 8(c)).

## 4. Discussion

Colorectal cancer (COAD) is the third most dangerous cancer taking approximately 700,000 lives every year worldwide [20]. Surgical removal is the primary treatment option, supplemented with 5-fluorouracil (5-FU) chemotherapy. Recently, the five-year survival rate of the patients has increased due to the administration of the various immunotherapies as alternative treatments for COAD [21, 22]. Previous studies have found that the use of *PD-1*, *PD-L1*, and *CTLA4* inhibitors can have a better immune effect on refractory (MSI-H and MSS) colorectal tumors [23, 24], but the recurrence and adverse reactions have been reported in the number of patients. Therefore, identifying meaningful immune targets in COAD could potentially improve the treatment outcomes of immunotherapy.

According to the ratio of immune cell infiltration in the COAD tumor microenvironment, we divided tumors into hot and cold tumors. By comparing hot tumors and cold tumors, we found many differences in immune-related factors between the two groups, which may explain the effect of different treatments or different patients' clinical histories. Human leukocyte antigen C (*HLA-C*), cytotoxic T-lymphocyte-associated antigen 4 (*CTLA4*), and natural killer (*NK*) cells were significantly elevated in hot tumors. HLA-C is a polymorphic membrane protein encoded by the HLA-C gene in class I major histocompatibility complex. HLA-C can promote inflammation by presenting antigen to T cells. In addition, HLA-C can activate NK cells to exert an innate immune response [25, 26]. Mostly, immunotherapeutic agents suppress the immune checkpoints to inhibit tumor growth. CTLA4 blockers have clinically been proven to improve the prognosis of cancer patients [27].

We performed differential analysis on the gene expressing in different samples of cold and hot tumors and then further used LASSO regression analysis and selected 12 hub genes (*CLK3*, *CYSLTR2*, *GJA10*, *CYP4Z1*, *FAM185A*, *LINC00324*, *EEF1A1P34*, *EEF1B2P8*, *PTCSC3*, *MIR6780A*, *LINC01666*, and *RNU6.661P*). CDC-like kinase 3 (*CLK3*) is a dual-specificity kinase that functions on substrates containing serine/threonine and tyrosine and is significantly upregulated in cholangiocarcinoma (CCA) and affecting the prognosis of patients through inhibiting purine metabolism [28]. In addition, hepatocellular carcinoma (HCC) [29], pancreatic cancer [30], and other cancers have become significant prognostic markers. The gap junction *α* (GJA) family has been demonstrated to be involved in the cellular proliferation and metastasis of gastric cancer and breast cancer [31, 32]. In the current study, we found that *CYSLTR2* [33, 34], *CYP4Z1* [35], *LINC00324* [36], and *PTCSC3* [37] have shown a close relationship with the initiation and development of tumors and also with the prognosis of the COAD patients.

Furthermore, we explored the relationship between 12 hub genes and immunity. After screening online immune databases, we identified cysteinyl leukotriene receptor 2 (*CYSLTR2*) as a possible immune-related marker. Overall, it has low expression in colon cancer as compared to adjacent normal tissues, suggesting it is a tumor suppressor gene. The expression of *CYSLTR2* has also significantly been downregulated in multiple myeloma [38], melanoma [39], and colorectal cancer [34], which reveals its tumor suppressor function in other cancers also. In COAD, dysregulation of CYSLTR2 has been associated with the proliferation and migration of the cancer cells [40]. Previous studies have shown that the increase in B cells in malignant tumors may be associated with differential miRNA expression [41, 42], and *CYSLTR2* showed a positive association with miR-125b in multiple myeloma (MM) [38] and causing abnormal infiltration of B cells in tumors. In the current study, we also found a positive correlation between the *CYSLTR2* and the expression of miR-125b in the colon cancer [43]. Moreover, *CYSLTR2* also participates in the polarization of M2 macrophages [44], and by associating with leptin, it promotes the proinflammatory activity of mast cells [45]. In the TIMER online database, we found a significant correlation of *CYSLTR2* with three common immune checkpoints (*PDCD1*, *CD274*, and *CTLA 4*) and immune cells (B cell, CD8+ T cell, CD4+ T cell, macrophage, neutrophil, and dendritic cells). These results suggest that *CYSLTR2* may regulate the adaptive immunity in COAD.

## 5. Conclusions

In the current study, we classified the COAD tumors based on their immune-related characteristics, as tumors with high immune cell infiltration (hot) and low immune cell infiltration (cold). The differentially expressed genes among hot and cold tumors were analyzed and constructed a prognostic model. Out of the total of 12 hub genes, *CYSLTR2* was selected as the potential immune target, which was further found to have a strong correlation with macrophages, CD8+ T cells, dendritic cells, and other immune cells. However, these results still need further experimental validation, we suggest *CYSLTR2* as a promising immune-related prognostic marker in COAD and could potentially be used as an important target in immunotherapy.

## Data Availability

The data used in this study is freely available in TCGA (http://tcga.cancer.gov/dataportal) portals. Our analyses' protocols and raw figures or other information related to our study could be asked from the corresponding author on reasonable request.

## Abbreviations

TCGA: The Cancer Genome Atlas project
LASSO: Least Absolute Shrinkage and Selection Operator
ROC: Receiver operating characteristic curve
AUC: Area under the curve
ssGSEA: Single-sample gene set enrichment analysis
DEGs: Differentially expressed genes
TP: Tumor purity
ES: ESTIMATE score
IS: Immune score
SS: Stromal score.
